# Role of polymorphic bile salt export pump (BSEP, *ABCB11*) transporters in anti-tuberculosis drug-induced liver injury in a Chinese cohort

**DOI:** 10.1038/srep27750

**Published:** 2016-06-13

**Authors:** Ru Chen, Jing Wang, Shaowen Tang, Yuan Zhang, Xiaozhen Lv, Shanshan Wu, Zhirong Yang, Yinyin Xia, Dafang Chen, Siyan Zhan

**Affiliations:** 1 Department of Epidemiology and Biostatistics, School of Public Health, Peking University Health Science Centre, Beijing, China; 2 Department of Epidemiology, School of Public Health, Nanjing Medical University, Nanjing, China; 3 Department of Clinical Epidemiology and Biostatistics, McMaster University, Hamilton, Canada; 4 Clinical Research Division, Peking University Institute of Mental Health, and Key Laboratory for Mental Health, Ministry of Health, Beijing, China; 5 Center for Tuberculosis Control and Prevention, Chinese Center for Disease Control and Prevention, Beijing, China

## Abstract

Evidence indicates that the polymorphisms in bile salt export pump (BSEP, encoded by *ABCB11*) may play an important role in the development of anti-tuberculosis drug-induced liver injury (ATDILI) and we aim to investigate the association between genetic variants of *ABCB11* and the risk of ATDILI in a Chinese cohort. A total of 89 tuberculosis patients with ATDILI and 356 matched ATDILI -free patients constituted cases and controls. Genetic polymorphisms of *ABCB11* were determined by TaqMan single-nucleotide polymorphism (SNP) genotyping assay. Odds ratio (OR) with 95% confidence intervals (CIs) was estimated by conditional logistic regression model. There were no significant differences in genotype frequencies of *ABCB11* between cases and controls. In the subgroup analysis, polymorphisms of rs2287616 were found to be associated with cholestatic/mixed pattern of liver injury under dominant and addictive model (OR = 3.84, 95% CI:1.16–12.75, *P* = 0.028 and OR = 2.51, 95% CI:1.12–5.62, *P* = 0.025, respectively), however the significance disappeared after Bonferroni correction. This study suggested that genetic variants of *ABCB11* gene might contribute to anti-tuberculosis drug-induced cholestatic liver injury in Chinese patients. Studies in larger, varied populations are required to confirm these findings.

The bile salt export pump (BSEP) which mainly located at the canalicular membrane of hepatocyte is an ATP-binding cassette (ABC) transporter encoded by the *ABCB11* gene. BSEP mediates the canalicular excretion of numerous bile salt into bile, which is regarded as the rate-controlling step of the vectorial transport of bile acids through hepatocytes^1^.

BSEP plays a key role in many liver diseases due to its crucial location and the important effects of bile acids^2^. Mutations of BSEP are known to cause cholestatic liver diseases of varying severity including progressive familial intrahepatic cholestasis^3^,^4^,^5^, benign recurrent intrahepatic cholestasis^6^,^7^ and intrahepatic cholestasis of pregnancy^8^,^9^, as well as drug-induced liver injury (DILI)^10^,^11^.

As a rare but potentially serious and idiosyncratic adverse drug reaction, DILI has been proven to be associated with genetic polymorphisms in genes involving in drug metabolism and transport, immune reaction and antioxidant response^12^. However, the association between genetic variations of *ABCB11* and risk of DILI is still unclear with inconsistent results from previous studies. Lang *et al*. found that polymorphism in rs2287622 was associated with drug-induced cholestasis in Caucasian patients^11^ while Kagawa *et al*. reported no contribution of the same polymorphism in Japanese patients with drug-induced cholestasis^13^. Furthermore, the study of Ulzurrun *et al*. showed that the C allele in the *ABCB11* rs2287622 polymorphism was associated with increased risk of hepatocellular type DILI for drugs containing a carbocyclic system with aromatic rings^10^.

As one of the most prevalent DILI, anti-tuberculosis drugs (ATDs) induced liver injury (ATDILI) is of importance due to relatively high incidence and large disease burden of tuberculosis (TB), which posed a considerable challenge to clinicians and researchers. The mechanism of ATDILI is poorly understood and whether genetic variants of *ABCB11* contribute to its susceptibility is seldom investigated. Vitro study has demonstrated that ATDs administration significantly down-regulated the expression of BSEP in liver^14^, therefore, it is reasonable to speculate that genetic variations in *ABCB11* may play a role in the development of ATDILI by altering the expression of BSEP and disturbing the transport of bile acids^15^. Therefore, the present study is aimed to explore the association between *ABCB11* polymorphisms and the risk of ATDILI in Chinese population.

## Results

### Baseline characteristics

89 patients with ATDILI and 356 matched controls were included in the study. Their baseline characteristics were summarized in Table 1. No significant difference was observed between the two groups regarding demographic parameters, including age, weight or body mass index, and baseline values of liver biochemical parameters (all *P* > 0.05). However, the peak AST, ALT, and TBIL levels were significantly higher in cases than those in controls during the treatment (*P* < 0.001).

### Genotype analysis

The observed genotype frequencies for the selected SNPs in the controls were all in Hardy-Weinberg equilibrium (*P* > 0.05, respectively) (Table 2). The genotype distributions of the four SNPs in *ABCB11* gene were presented in Table 3. None of them was significantly associated with the developmentof ATDILI.

### Subgroup analysis

According to diagnostic standard proposed in international consensus meeting, ATDILI could be categorized as cholestatic, mixed and hepatocellular liver injury based on the ratio of ALT and alkaline phosphatase (ALP). In this study, 28 cases with ALP data and were dichotomized into hepatocellular group or cholestatic/mixed group, with the rest of 61 cases classified as unclear type. The association between selected SNPs and the risk of ATDILI among different diagnoses of liver injury were shown in Table 4. The frequency of rs2287616 in *ABCB11* gene were significantly different between cholestatic/mixed patients and matched controls under dominant model and addictive model (OR = 3.84, 95% CI:1.16–12.75, *P* = 0.028 and OR = 2.51, 95% CI:1.12–5.62, *P* = 0.025, respectively), however the significance disappeared after Bonferroni correction.

## Discussion

In this study, we investigated the association between polymorphisms of *ABCB11* and the development of ATDILI among Chinese population. The results shows that though there was no significant differences in genotype frequencies between patients with and without ATDILI in general, the polymorphisms of rs2287616 in *ABCB11* gene were significantly associated with cholestatic/mixed liver injury in the subgroup analysis. Besides, patients carrying the CC genotype of rs2287616 had a higher value of TBIL compared with CT/TT genotype. In addition, significantly increased frequencies of rs2287616 CC genotype were noticed in some adverse effects symptoms including gastrointestinal disorders, arthralgia and pruritus. These findings suggested that the polymorphisms of *ABCB11* gene might be associated with ATDs-induced cholestatic liver injury in Chinese TB patients.

BSEP is responsible for the bile salt-dependent bile flow and is regarded as an important gene in many liver diseases including DILI^2^. Previous studies have identified a common BSEP polymorphism V44A (1331T > C, rs2287622) as genetic risk factor for DILI in Caucasian^10^,^11^ In the present study, we tested the same SNP in Chinese patients, however, no significant association was found between rs2287622 polymorphism and the development of DILI, which was similar to the study conducted in Japan^13^. Since genetic associations were ethnicity-specific and drug-specific, the association of *ABCB11* rs2287622 genetic variations and DILI should be investigated in other populations and other drugs.

Despite of rs2287622, a new SNP, rs2287616 was found to be associated with cholestatic/mixed liver injury in this study. Limited by the sample size of cholestatic pattern of ATDILI, supplementary analyses were taken to provide indirect evidence to the association. The signs and symptoms of cholestatic liver disease may include hyperbilirubinemia, gastrointestinal disorders, fatigue, pruritus, arthralgia, and so on. Therefore, we compare the value of liver function tests and the symptoms of common adverse drug effects in ATDILI patients with different genotypes of *ABCB11* rs2287616. The results showed that polymorphisms in rs2287616 had a significant association with increased TBIL, gastrointestinal disorders, arthralgia and itching/rash, indicating the role of genetic variations of *ABCB11* in the development of ATDs-induced cholestatic liver injury. Besides, supplementary analyses revealed the association between rs496550 and an evaluated TBIL as well as the symptom of nausea/vomiting, which pointed the importance of *ABCB11* polymorphisms in ATDs-induced adverse reaction.

The *ABCB11* gene encoding human BSEP consists of 28 exons (including non-coding exon 1) and spans approximately 108 kb on the long arm of chromosome 2(2q24–31)^16^ Rs2287616 is a synonymous mutation on exon 9 and rs496550 is located on 3′URT. Though the function and mechanism of these two SNPs is poorly understood, as they are both tag SNPs that represent a group of SNPs with high linkage disequilibrium in regions of *ABCB11* gene, it indicates a possible role of genetic variations within the blocks of *ABCB11* in ATDs-induced cholestatic liver injury. Moreover, vitro study has proven that INH/RMP administration significantly down-regulated the expression of BSEP in liver of mice^14^. Therefore, the genetic susceptibility to ATDILI may be explained by the decreased expression of BSEP in the presence of polymorphisms in *ABCB11*^17^, leading to increased bile salt concentrations in hepatocytes, which contributes to the development of liver injury.

The emerging role of hepatic transporters in regulating the movement of endogenous and exogenous chemicals (e.g., bile acids and drugs) across cellular and tissue membranes is critical in determining the pathophysiology of liver disease^18^. In this study, we focus on the gene that regulates the transport of bile acids. The different findings in subgroup analysis pinpointed the significant contribution of BSEP to the complexity of ATDILI. The mechanism of ATDILI varies among different patterns of liver injury, which consequently changes the genetic susceptibility to the disease. Future investigations should pay attention to the specific mechanism of each pattern and evaluate the genetic association respectively.

The major strength of the present study is that it was a case-control study nested in the ACADS cohort, which meant this study was less likely to be subjected to recall bias and other possible biases introduced by self-selection of patients. Secondly, each case was verified by experts strictly to minimize the diagnosis misclassification. In addition, we performed a 1:4 matching to control effects of some potential confounders as well as to increase the statistical power.

However, the results of this study are limited by the small sample size in cholestatic/mixed patients. Because the information of ALP was not included for all patients, only a few cases could be identified as hepatocellular, cholestatic or mixed. As a result, we have to combine cholestatic group and mixed group together in the subgroup analysis, and take supplementary analyses to further explain the results. Besides, the ATDs were used as a combination, thus it is difficult to identify the association of each drug and its adverse reaction.

In conclusion, we found that genetic polymorphism of *ABCB11* might contribute to ATDs-induced cholestatic liver injury in Chinese population. Studies in larger, varied populations are required to confirm these findings.

## Methods

### Study population

Anti-TB treatment patients were recruited from ADACS cohort^19^, which is a prospective longitudinal study of ATD induced adverse reactions based on community population. From October 2007 to June 2008, a total of 4488 newly diagnosed TB patients were recruited from four provinces in China. All patients were treated with a standard chemotherapy regimen with a combination of ATDs including isoniazid (INH), rifampicin (RMP), pyrazinamide (PZA), ethambutol (EMB) and/or streptomycin (SM) for six to nine months^20^. Liver function tests were performed within 2 months of the initiation of treatment in all patients or whenever the patients exhibited symptoms of suspected hepatitis (e.g. anorexia, nausea, vomiting, malaise, teacoloured urine). A total of 4304 patients finished the follow-up. This study was approved by the Ethics Committee of Center for Tuberculosis Control and Prevention of China and Health Science Center of Peking University. Written informed consent was obtained from every participant or surrogate before enrolment. The present study was conducted in accordance with the Declaration of Helsinki Principles.

ATDILI was defined as: (i) an increase in alanine aminotransferase (ALT) levels greater than two-times of the upper limit of normal (ULN) with/without a combined increase in aspartate aminotransferase (AST) and total bilirubin (TBIL) levels provided one of them was greater than two-times of ULN during the treatment^21^; (ii) causality assessment result was certain, probable or possible based on the WHO Uppsala Monitoring Center system^22^. All suspected hepatitis patients were reviewed by experts from Chinese State Food and Drug Administration. Patients with any of the following were excluded from the present study: (i) abnormal serum ALT, AST or TBIL levels before anti-TB treatment; (ii) carriers of the hepatitis B or C virus; (iii) alcoholic liver disease or habitual alcohol drinking; (iv) the concomitant use of hepatotoxic drugs; and (v) a history of chronic liver disease or systemic diseases that may cause liver dysfunction. Among the remained patients, those fulfilled the criteria of ATDILI were assigned into the case group. For each ATDILI case, four controls were randomly selected and matched with age (within 5 years old), sex, treatment history, disease severity, drug dosage and place of sample collection.

### Selection of SNPs

A tag-SNP approach was used to select the SNPs. All eligible SNPs in the gene region including 2-kb upstream were obtained from the Chinese Han population data available on the International HapMap website (http://hapmap.ncbi.nlm.nih.gov, HapMap Genome Browser release #24). The tag SNPs were selected using Haploview 4.2 software based on the following criteria: i) minor allele frequency more than 0.10; ii) r^2^ of pairwise linkage disequilibrium more than 0.80. As a result, four SNPs (rs496550, rs2287622, rs3770592 and rs2287616) were chosen for genotyping (Table 2).

### Laboratory procedures

Blood sample was collected from each patient during laboratory examination after their recruitment. Then DNA was extracted and genotyped by TaqMan allelic discrimination technology on the ABI 7900 Real-Time PCR System (Applied Biosystems, Foster City, CA)^23^. The primers and probes for each SNP were designed by Nanjing Steed BioTechnologies Co., Ltd. Genotyping was performed by blinding the case or control status, and with a positive control of a DNA sample with a known heterozygous genotype in each test. More than 10% of the samples were repeated using the same assay and the results were 100% concordant. The overall call rate of genotyping was more than 98%.

### Statistical analysis

Continuous variables were described as mean ± standard deviation (SD) or median (IQR, inter-quartile range) and differences between groups were analyzed by two-factor analysis of variance test or non-parametric test. Categorized variables were described as percentage and analyzed using the χ^2^ test. Hardy-Weinberg equilibrium was tested by a goodness-of-fit χ^2^ test. Genotype frequencies were compared in the ATDILI patient and control groups by multivariate conditional logistic regression analysis, with weight and usage of liver-protective drug as covariates. Kruskal-Waillis test was used to compare the distribution of mean AST, ALT and TBIL values in the different genotype patterns. The associations between selected SNPs and the risk of ATDILI were estimated by odds ratios (OR) with 95% confidence intervals (CIs). Three different analysis models, dominant (Dom), recessive (Rec) and addictive (Add), were used to compare genotype frequencies. A two-tailed P-value less than 0.05 was considered to be statistically significant. Considering the potential false positive rate incurred by multiple comparisons of SNPs, we applied the Bonferroni correction method to adjust the P value. The statistical analyses were performed with SPSS for Windows (version 19.0, IBM Inc.).

## Additional Information

**How to cite this article**: Chen, R. *et al*. Role of polymorphic bile salt export pump (BSEP, ABCB11) transporters in anti-tuberculosis drug-induced liver injury in a Chinese cohort. *Sci. Rep.* **6**, 27750; doi: 10.1038/srep27750 (2016).

## Supplementary Material

Supplementary Information

## Figures and Tables

**Figure 1:**
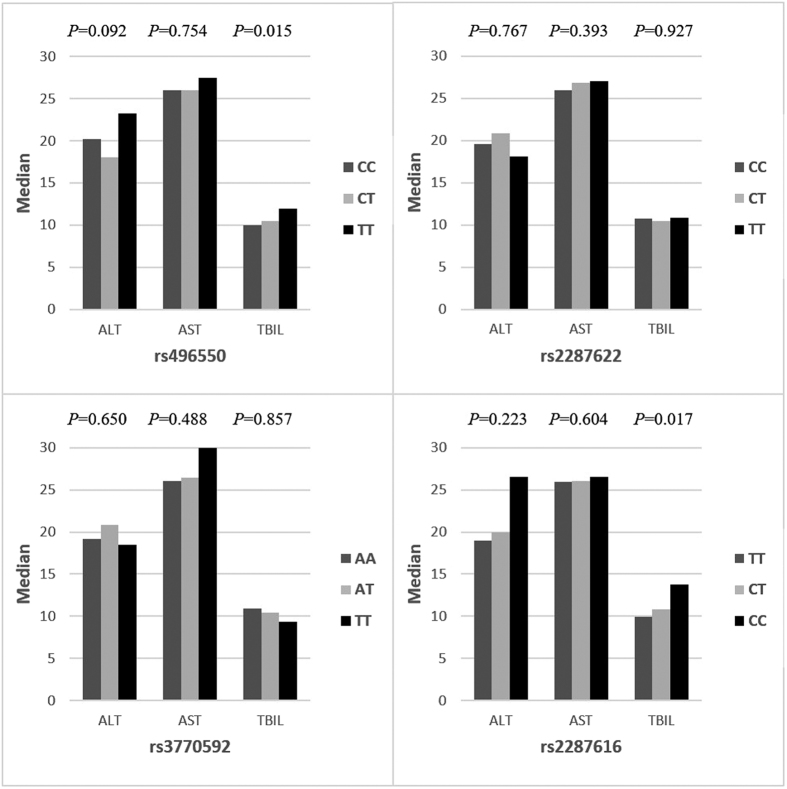
Median values of ALT, AST and TB in different genotype groups of *ABCB11* gene. The values are shown as U/L for ALT and AST and μmol/L for TBIL. Abbreviations: ALT, alanine aminotransferase; AST, aspartate aminotransferase; TBIL, total bilirubin.

**Table 1:** Characteristics of patients with and without ATDILI.

Characteristic	Patients with ATDILI(N = 89)	Patients without ATDILI(N = 356)	P value
Sex (male/female)	65/24	260/96	
Treatment history (primary/re-treatment)	78/11	312/44	
Age (years)^a^	43.7 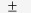 16.4(20.0–80.0)	43.6 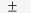 16.4(17.0–84.0)	0.742^c^
Weight (kg)^a^	53.2 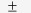 8.0(38.0–80.0)	53.5 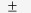 7.4(31.0–84.0)	0.748^c^
BMI (kg/m^2^)^a^	19.5 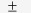 2.3(14.0–27.7)	19.4 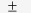 2.3(13.5–27.0)	0.959^c^
Baseline value			
AST(U/L)^b^	24.8(17.2–32.6)	21.4(15.3–27.0)	0.283^d^
ALT(U/L)^b^	16.9(10.8–26.3)	16.0(10.4–22.0)	0.087^d^
TBIL(μmol/L)^b^	9.5(7.5–13.7)	9.7(7.4–12.5)	1.000^d^
during treatment (peak value)			
AST(U/L)^b^	95.1(60.6–174.7)	23.7(16.7–29.0)	< 0.001^d^
ALT(U/L)^b^	121.0(88.2–183.6)	17.0(11.6–23.2)	< 0.001^d^
TBIL(μmol/L)^b^	14.3(11.4–18.0)	9.7(6.8–13.7)	< 0.001^d^

Abbreviations: BMI, body mass index; ALT, alanine aminotransferase; AST, aspartate aminotransferase; TBIL, total bilirubin.

^a^Values are presented as mean ± SD(range).

^b^Values are presented as median (inter-quartile range).

^c^two-factor analysis of variance test.

^d^Median test.

**Table 2:** Information on four genotyped tag SNPs in *ABCB11* gene.

SNP	Chromosome position^a^	Position	SNP name	MAF^b^	HWE p-value^c^
rs496550	2: 168923202	3′ UTR	420A > G	34.9	0.472
rs2287622	2: 168973818	Exon	1331T > C	32.1	0.415
rs3770592	2: 168981530	Intron	1084–1551A > T	23.2	0.867
rs2287616	2: 168990902	Exon	807T > C	38.4	0.265

^a^NCBI SNP database on GRCh37.p10 assembly.

^b^MAF(Minor Alleles Frequency) for Han Chinese in Beijing in the HapMap database.

^c^Hardy–Weinberg equilibrium (HWE) P-value in the control group.

**Table 3:** *ABCB11* polymorphisms in patients with and without ATDILI.

Genotype	Cases N(%)	Controls N(%)	OR (95% CI)	P value	Model	OR (95% CI)	P value
rs496550							
GG	38(43.7)	150(43.4)	1 (reference)		Dom	0.95(0.60–1.53)	0.844
AG	38(43.7)	151(43.6)	0.99(0.60–1.64)	0.965	Rec	0.85(0.42–1.76)	0.668
AA	11(12.6)	45(13.0)	0.85(0.40–1.81)	0.673	Add	0.94(0.67–1.32)	0.727
rs2287622							
CC	44(50.0)	185(52.3)	1 (reference)		Dom	1.06(0.67–1.68)	0.810
CT	37(42.0)	146(41.2)	1.02(0.63–1.65)	0.925	Rec	1.31(0.53–3.21)	0.557
TT	7(8.0)	23(6.5)	1.32(0.53–3.33)	0.553	Add	1.09(0.75–1.58)	0.666
rs3770592							
AA	54(60.7)	217(61.3)	1 (reference)		Dom	1.02(0.63–1.64)	0.949
AT	32(36.0)	121(34.2)	1.04(0.64–1.71)	0.862	Rec	0.78(0.23–2.69)	0.692
TT	3(3.3)	16(4.5)	0.79(0.23–2.77)	0.714	Add	0.98(0.66–1.47)	0.931
rs2287616							
TT	37(43.0)	178(50.3)	1 (reference)		Dom	1.31(0.81–2.11)	0.266
CT	38(44.2)	152(42.9)	1.18(0.71–1.97)	0.532	Rec	1.88(0.86–4.08)	0.112
CC	11(12.8)	24(6.8)	2.01(0.90–4.49)	0.090	Add	1.33(0.93–1.90)	0.123

Abbreviations: Dom, dominant model; Rec, recessive model; Add, additive model.

**Table 4:** *ABCB11* polymorphisms in patients with and without ATDILI among different diagnoses of liver injury.

SNP	Model	Hepatocellular (N = 11)		Cholestatic/Mixed(N = 17)		Unclear (N = 61)	
		OR(95%CI)	P value	OR(95%CI)	P value	OR(95%CI)	P value
rs496550	Dom	0.53(0.14–1.98)	0.345	1.53(0.49–4.74)	0.461	0.93(0.53–1.66)	0.814
	Rec	0.66(0.06–7.11)	0.731	3.08(0.57–16.73)	0.193	0.67(0.28–1.61)	0.368
	Add	0.64(0.23–1.79)	0.394	1.61(0.70–3.70)	0.263	0.88(0.58–1.33)	0.538
rs2287622	Dom	2.12(0.57–7.87)	0.262	0.64(0.23–1.80)	0.402	1.08(0.61–1.91)	0.803
	Rec	3.56(0.20–64.34)	0.391	0.48(0.05–4.23)	0.506	1.59(0.53–4.77)	0.411
	Add	2.14(0.67–6.83)	0.199	0.68(0.30–1.55)	0.357	1.14(0.71–1.82)	0.594
rs3770592	Dom	1.02(0.25–4.16)	0.975	0.94(0.31–2.89)	0.911	1.06(0.60–1.88)	0.848
	Rec	NA	NA	NA	NA	NA	NA
	Add	0.85(0.24–3.07)	0.807	0.83(0.31–2.20)	0.702	1.08(0.66–1.75)	0.769
rs2287616	Dom	0.35(0.07–1.67)	0.186	3.84(1.16–12.75)	0.028	1.17(0.66–2.07)	0.590
	Rec	NA	NA	3.27(0.68–15.73)	0.140	1.85(0.71–4.79)	0.206
	Add	0.36(0.08–1.62)	0.181	2.51(1.12–5.62)	0.025	1.24(0.80–1.94)	0.335

Abbreviations: Dom, dominant model; Rec, recessive model; Add, additive model; NA, not applicable.

**Table 5:** Distribution of *ABCB11* rs2287616 genotype in ATDILI patients with various symptoms.

		Genotype distribution			χ^2^	P value
Symptoms		TT N(%)	CT N(%)	CC N(%)		
Fever	Yes	2(28.6)	4(57.1)	1(14.3)	1.093	0.579
	No	23(47.9)	18(37.5)	7(14.6)		
Dizziness/headache	Yes	9(37.5)	11(45.8)	4(16.7)	1.087	0.581
	No	16(51.6)	11(35.5)	4(12.9)		
Gastrointestinal disorders^a^	Yes	5(26.3)	8(42.1)	6(31.6)	8.162	0.017
	No	20(55.6)	14(38.9)	2(5.6)		
Nausea/ vomiting	Yes	15(38.5)	19(48.7)	5(12.8)	4.264	0.119
	No	10(62.5)	3(18.8)	3(18.8)		
Fatigue/lethargy/insomnia	Yes	6(30.0)	9(45.0)	5(25.0)	4.209	0.122
	No	19(54.3)	13(37.1)	3(8.6)		
Arthralgia	Yes	1(10.0)	5(50.0)	4(40.0)	9.130	0.010
	No	24(53.3)	17(37.8)	4(8.9)		
Pruritus	Yes	6(31.6)	7(36.8)	6(31.6)	7.092	0.029
	No	19(52.8)	15(41.7)	2(5.6)		

^a^Include diarrhea, abdominal pain and abdominal distension period.
