# Supplementary material for: Role of polymorphic bile salt export pump (BSEP, ABCB11) transporters in anti-tuberculosis drug-induced liver injury in a Chinese cohort
Source: Sci Rep. 2016 Jun 13;6:27750. doi: 10.1038/srep27750 (PMC4904206; doi:10.1038/srep27750)
Supplement: Supplementary Information [file srep27750-s1.pdf]

Role of polymorphic bile salt export pump (BSEP, *ABCB11*) transporters in anti-tuberculosis drug-induced liver injury in a Chinese cohort

Ru Chen, Jing Wang, Shaowen Tang, Yuan Zhang, Xiaozhen Lv, Shanshan Wu, Zhirong Yang, Yinyin Xia, Dafang Chen, Siyan Zhan*

Supplementary Table S1 Distribution of *ABCB11* rs496550 genotype in ATDILI patients with various symptoms

*Include diarrhea, abdominal pain and abdominal distension

Symptoms		Genotype distribution			χ^2	P value
		GG N(%)	AG N(%)	AA N(%)		
Fever	Yes	1(14.3)	5(71.4)	1(14.3)	3.032	0.220
	No	24(49.0)	20(40.8)	5(10.2)		
Dizziness/ headache	Yes	8(33.3)	13(54.2)	3(12.5)	2.182	0.336
	No	17(53.1)	12(37.5)	3(9.4)		
Gastrointestinal disorders*	Yes	9(47.4)	7(36.8)	3(15.8)	1.131	0.568
	No	16(43.2)	18(48.6)	3(8.1)		
Nausea/ vomiting	Yes	12(30.8)	22(56.4)	5(12.8)	10.056	0.007
	No	13(76.5)	3(17.6)	1(5.9)		
Fatigue/lethargy/ insomnia	Yes	11(55.0)	6(30.0)	3(15.0)	2.775	0.250
	No	14(38.9)	19(52.8)	3(8.3)		
Arthralgia	Yes	5(45.5)	4(36.4)	2(18.2)	0.925	0.630
	No	20(44.4)	21(46.7)	4(8.9)		
Pruritus	Yes	11(55.0)	7(35.0)	2(10.0)	1.410	0.494
	No	14(38.9)	18(50.0)	4(11.1)		

Supplementary Table S2 Distribution of *ABCB11* rs2287622 genotype in ATDILI patients with various symptoms

*Include diarrhea, abdominal pain and abdominal distension

Symptoms		Genotype distribution			χ^2	P value
		CC N(%)	CT N(%)	TT N(%)		
Fever	Yes	5(71.4)	1(14.3)	1(14.3)	2.730	0.255
	No	24(48.0)	23(46.0)	3(6.0)		
Dizziness/ headache	Yes	14(58.3)	9(37.5)	1(4.2)	1.142	0.565
	No	15(45.5)	15(45.5)	3(9.1)		
Gastrointestinal disorders*	Yes	11(57.9)	7(36.8)	3(5.3)	0.588	0.745
	No	18(47.4)	17(44.7)	3(7.9)		
Nausea/	Yes	20(50.0)	18(45.0)	2(5.0)	1.065	0.587

vomiting	No	9(52.9)	6(35.3)	2(11.8)		
Fatigue/lethargy/	Yes	9(45.0)	9(45.0)	2(10.0)	0.661	0.719
insomnia	No	20(54.1)	15(40.5)	2(5.4)		
Arthralgia	Yes	8(72.7)	3(27.3)	0(0.0)	2.948	0.229
	No	21(45.7)	21(45.7)	4(8.7)		
Pruritus	Yes	13(65.0)	5(25.0)	2(10.0)	3.739	0.154
	No	16(43.2)	19(51.4)	2(5.4)		

Supplementary Table S3 Distribution of *ABCB11* rs3770592 genotype in ATDILI patients with various symptoms

*Include diarrhea, abdominal pain and abdominal distension

Symptoms		Genotype distribution			χ^2	P value
		AA N(%)	AT N(%)	TT N(%)		
Fever	Yes	5(71.4)	1(14.3)	1(14.3)	2.076	0.354
	No	31(62.0)	17(34.0)	2(4.0)		
Dizziness/	Yes	14(58.3)	8(33.3)	2(8.3)	0.936	0.626
headache	No	22(66.7)	10(30.3)	1(3.0)		
Gastrointestinal	Yes	12(63.2)	7(36.8)	0(0.0)	1.750	0.417
disorders*	No	24(63.2)	11(28.9)	3(7.9)		
Nausea/	Yes	25(62.5)	13(32.5)	2(5.0)	0.063	0.969
vomiting	No	11(64.7)	5(29.4)	1(5.9)		
Fatigue/lethargy/	Yes	12(60.0)	7(35.0)	1(5.0)	0.167	0.920
insomnia	No	24(64.9)	11(29.7)	2(5.4)		
Arthralgia	Yes	10(90.9)	1(9.1)	0(0.0)	4.562	0.102
	No	26(56.6)	17(37.0)	3(6.5)		
Pruritus	Yes	15(75.0)	4(20.0)	1(5.0)	1.996	0.369
	No	21(56.8)	14(37.8)	2(5.4)		
